# Engineering the Electronic Structure and Optoelectronic Properties of Chiral Metal Halides through Cation Design

**DOI:** 10.1021/acsmaterialslett.5c00666

**Published:** 2025-07-18

**Authors:** Clarissa Coccia, Marco Moroni, Massimo Boiocchi, Marta Morana, Maddalena Patrini, Doretta Capsoni, Alessio Porta, Andera Olivati, Giulia Folpini, Annamaria Petrozza, Luca Gregori, Edoardo Mosconi, Filippo De Angelis, Lorenzo Malavasi

**Affiliations:** † Department of Chemistry and INSTM, 19001 University of Pavia, Via Taramelli 12, 27100, Pavia, Italy; ‡ Centro Grandi Strumenti, 19001 University of Pavia, Via Bassi 21, 27100, Pavia, Italy; § Center for Nano Science and Technology@PoliMi, Istituto Italiano di Tecnologia, 20134, Milan, Italy; ∥ Institute for Photonics and Nanotechnology, CNR − IFN, 20133, Milano, Italy; ⊥ Department of Physics, 19001 University of Pavia, Via Bassi 6, 27100, Pavia, Italy; # Department of Chemistry, Biology and Biotechnology, 9309 University of Perugia, Via Elce di Sotto 8, 06123, Perugia, Italy; g Computational Laboratory for Hybrid/Organic Photovoltaics (CLHYO), Istituto CNR di Scienze e Tecnologie Chimiche “Giulio Natta” (CNR-SCITEC), Via Elce di Sotto 8, 06123, Perugia, Italy; h Chemistry Department, College of Science, King Saud University, 11451, Riyadh, Saudi Arabia; i SKKU Institute of Energy Science and Technology (SIEST), Sungkyunkwan University, Suwon 440-746, South Korea

## Abstract

The tunability of hybrid organic–inorganic metal halides through targeted chemical design is one of their most attractive features, enabling fine control over physical properties for optoelectronic applications. In chiral systems, where chirality is introduced via organic amines, this tunability is often limited by the scarcity of suitable chiral cations. In this study, we report a family of 1D lead- and tin-based chiral hybrid halides incorporating a tailor-made cation bearing both amino and hydroxyl functional groups. This chiral ligand enables the synthesis of enantiopure (*S/R*-AMOL)­SnI_3_ and (*S/R*-AMOL)­PbI_3_, where *S/R*-AMOL stands for (2*S*,2′*S*)-1,1′-azanediylbis­(butan-2-ol) or (2*R*,2′*R*)-1,1′-azanediylbis­(butan-2-ol). These compounds exhibit distinctive structural arrangements and bonding interactions, demonstrating effective chirality transfer through chiral centers bearing hydroxyl groups. Remarkably, substantial differences in the electronic structure and chiroptical properties are observed between the Sn and Pb analogues, including variations in emission characteristics, exciton binding energy, and orbital contributions to the electronic structure.

## Graphical abstract

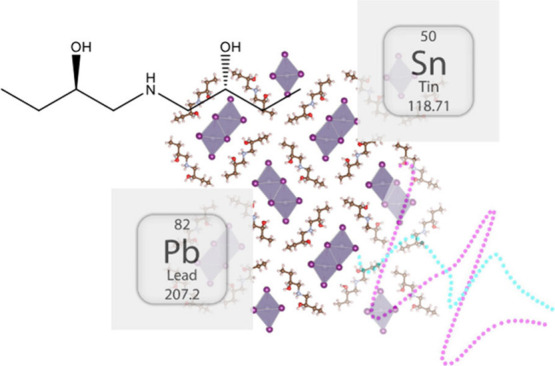

The emergence of chiral metal halide perovskites and related structures, generally defined as chiral metal halides, as an exciting field of research stems from both their inherent structural complexity and their remarkable potential for innovative technological applications.^1–4^ By introducing chirality into the inorganic framework, researchers have unlocked novel functionalities such as circular dichroism (CD), circularly polarized luminescence (CPL), second-harmonic generation (SHG), and topological quantum states. In addition, potential applications of chiral metal halides extend well beyond conventional optoelectronics. Their unique properties may also find use in spintronics, quantum computing, and emerging fields such as topological photonics.^3,5^ Understanding the underlying principles of chiral metal halides and their interactions with external stimuli will be vital in harnessing their full potential for future technologies.^5^

To date, all the chiral perovskites and perovskite derivatives reported are based on the few commercially available chiral amines, with methylbenzylamine, MBA, and its halogenated derivatives (X-MBA; X = F, Cl, Br) as the most widely used ligands.^2,6–9^ Based on these chiral cations, several 2D chiral perovskites have been prepared and investigated, helping in clarifying, for example, the role of the nature of halogen substituent and its position on the aromatic ring on the CD and CPL response due to the different extents of hydrogen bonding with the halide of the inorganic framework and the local distortion induced on the octahedra.^9–12^ Other common monoammonium chiral cations employed to date for 2D chiral perovskites involve 1-(2-naphthyl)­ethyl­ammonium (NEA), β-methylphenethyl­ammonium (MPA), and 1-(1-naphthyl)­ethylammonium (NPB), as well as samples with alloyed cations on the A-site of the perovskite.^13–16^ All these studies provided a set of tuning strategies of the chiroptical and of spin-based properties of chiral perovskites shedding light on the role of short-range and noncovalent intermolecular interactions, structural distortions, and Rashba splitting and build also the bases for device engineering and computational modeling.^17^

To widen the understanding of chirality in metal halide perovskites, unraveling unexplored properties, advance the optoelectronic devices including chiral metal halides, and gain a deeper comprehension of the chirality transfer mechanism and structure–property correlations in this emerging area, it is important to extend the set of available chiral cations and therefore of novel compositions and structural topologies. Such extension could also involve the design of multifunctional ligands where, in addition to the amine moiety, other functional groups are present. This is of particular interest in the field of chiral metal halides, where the structural framework and, in turn, the optoelectronic properties are strongly connected to the ability of the organic cation of short-range bonding (hydrogen and van der Waals) to the inorganic framework.^18–21^ Therefore, the design and use of chiral bifunctional ligands could widen the scope of chiral perovskite and perovskite derivative engineering. In addition, the comprehension of the role of the central metal nature on the chiroptical properties, together with the need of moving toward lead-free compositions, is another urgent issue in the field of chiral metal halides. To date, few reports explored metals other than Pb, and 2D perovskites or low-dimensional chiral metal halides incorporating Sn, Bi, Cu, or Ge have been reported in the past few years.^8,22–31^ The substitution of Pb with other metals allowed focusing on the role of spin–orbit coupling on chiroptical properties and showed, in most of the cases, an increased octahedral distortion leading to enhanced CD and second-order nonlinear responses.

To try to add an additional piece of information on both the role of the chiral cation and metal nature, we performed the synthesis of dimeric bifunctional chiral ligands, namely, (2*R*,2′*R*)-1,1′-azanediylbis­(butan-2-ol) and (2*S*,2′*S*)-1,1′-azanediylbis­(butan-2-ol), the structures of which are reported in Figure 1 for both the enantiomers. As can be seen, the chiral ligands contain one amino and two hydroxyl functional groups with the chiral carbons being those bearing the −OH groups. For the sake of brevity, in the following, the chiral ligands will be defined as “(*S*-/*R*-)­AMOL”, indicating the fact that, from a chemical point of view, such bifunctional molecules are amino alcohols (or amino diols).

**Figure 1:**
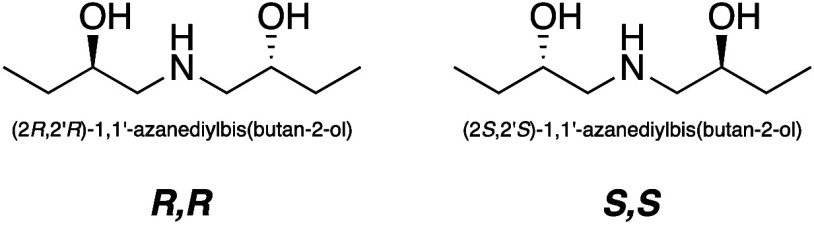
Structures of (2*R*,2′*R*)-1,1′-azanediylbis­(butan-2-ol) and (2*S*,2′*S*)-1,1′-azanediylbis­(butan-2-ol).

The two enantiopure dimeric AMOL cations, namely, (2*R*,2′*R*)-1,1′-azanediylbis­(butan-2-ol) and (2*S*,2′*S*)-1,1′-azanediylbis­(butan-2-ol), have been used to prepare lead and tin iodide samples by means of solution chemistry as reported in the Experimental Section (see the SI). For the tin-containing compositions, we could achieve the growth of high-quality single crystals of the *S*-enantiomer and those of lower quality for the *R*-enantiomer, which have been used to solve the structure through single-crystal X-ray diffraction (SC-XRD). Table 1 reports the main crystallographic data of the two enantiopure chiral tin iodides with formula (*S*/*R*-AMOL)­SnI_3_.

**Table 1:** Crystallographic Data of (*S*/*R*-AMOL)­BI_3_ (B = Sn, Pb) and Octahedral Distortion Parameters^a^

	**(** ***S*** **-AMOL)SnI**_**3**_	**(** ***R*** **-AMOL)SnI**_**3**_	**(** ***S*** **-AMOL)PbI**_**3**_	**(** ***R*** **-AMOL)PbI**_**3**_
Crystal system	Orthorhombic	Orthorhombic	Orthorhombic	Orthorhombic
Space group	*P*2_1_2_1_2_1_	*P*2_1_2_1_2_1_	*P*2_1_2_1_2_1_	*P*2_1_2_1_2_1_
*a* (Å)	4.5687(2)	4.573(2)	4.5935(4)	4.5936(5)
*b* (Å)	18.3063(6)	18.307(8)	18.263(1)	18.2644(8)
*c* (Å)	20.4374(6)	20.444(9)	20.629(1)	20.6146(8)
*V* (Å^3^)	1709.30(11)	1711.11(13)	1730.7(2)	1729.6(2)
*Z*	4	4	4	4
λ_oct_	1.0047	1.0047	1.0073	1.0067
*D*	0.0333	0.0337	0.037	0.0315
σ^2^	8.54	8.33	19.24	18.69
B–I lengths (Å)	2.9376(6)	2.939(3)	3.01866(0)	3.05647
	3.1562(6)	3.156(4)	3.21214(0)	3.17738
	3.1964(6)	3.195(4)	3.23187(0)	3.26298
	3.2057(6)	3.211(4)	3.33235(0)	3.31022
	3.2604(6)	3.261(4)	3.40022(0)	3.37109
	3.4642(6)	3.466(4)	3.46515(0)	3.43379

a CCDC code for (*S*-AMOL)­SnI_3_: 2365047.

From a structural point of view, the material consists of 1D double chains running along the crystallographic *a*-axis built up by face-sharing [SnI_6_]^4–^ octahedra, generating the structural motif reported in Figures 2a and 2b (referring to (*S*-AMOL)­SnI_3_). This is a quite rare structural arrangement in halide perovskites and perovskite derivatives and, to the best of our knowledge, has been previously observed only in one hybrid iodoplumbate-containing protonated urea as the organic cation.^32^ One crystallographically independent (*S*-)­AMOL molecule interacts with the iodide anions of the metal-halide chains through four short-distance bonds. In particular, the hydrogens of the protonated −NH_2_^+^ moiety display two bonds at 3.109(4) and 3.170(5) Å, while even shorter bonds are established between the H atoms of the −OH groups and the I of the octahedra: 3.001(4) and 2.831(4) Å (see Figure 2c). This is a complex bonding pattern involving both the amino and hydroxyl functional groups which, through this peculiar framework, gives the origin of the structural arrangement shown in Figure 2.

**Figure 2:**
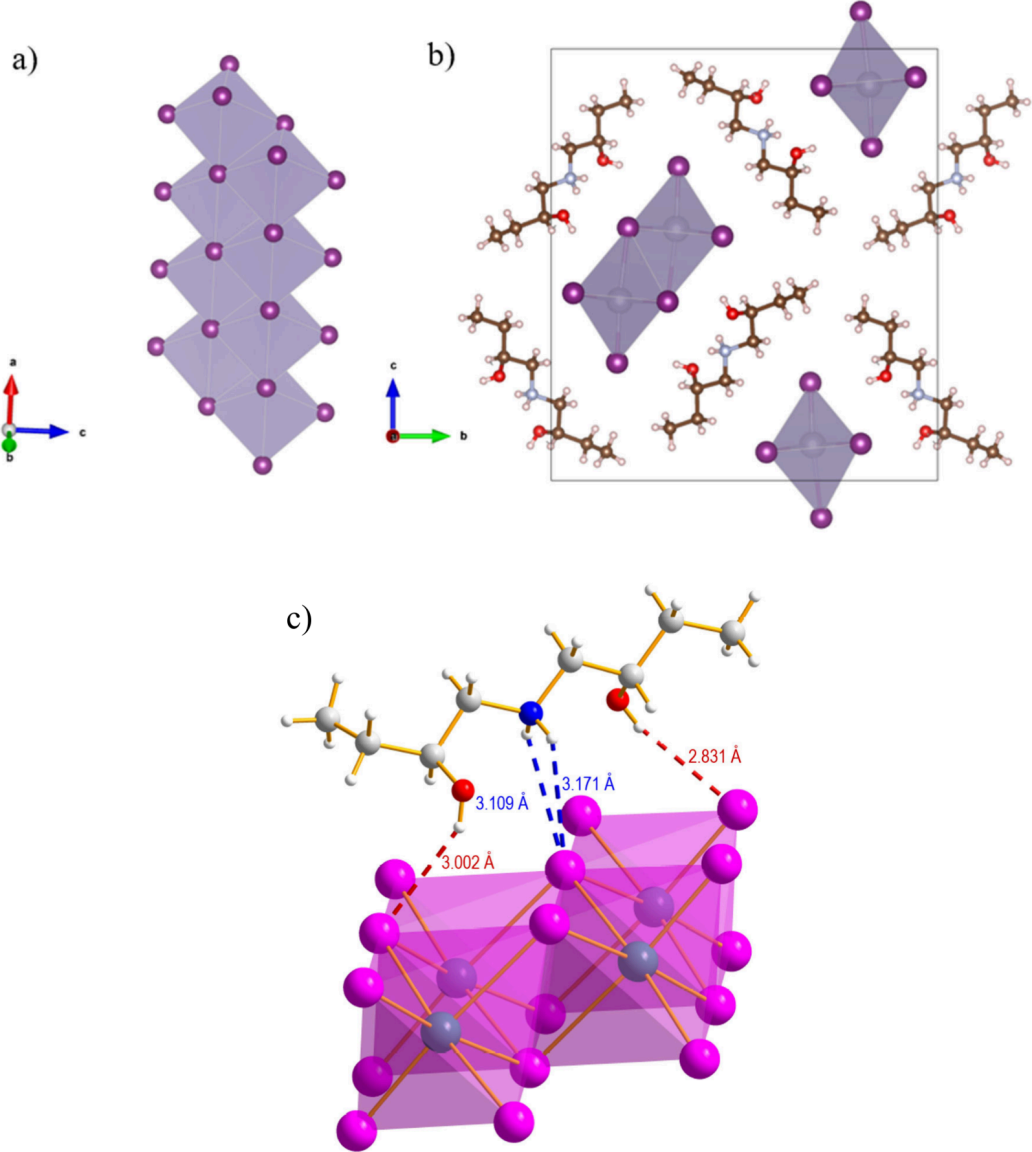
a) Representation of the 1D inorganic double chains of (*S*-AMOL)­SnI_3_ along the *a*-axis; b) sketch of the unit cell along the *c*-axis; c) detail of the crystal structure highlighting the interaction between the chiral cation and the inorganic framework.

There are few examples of perovskites or perovskite derivatives containing bifunctional organic spacers with −NH_2_ and −OH, namely, [(HOC_*n*_H_2*n*_NH_3_)_2_PbI_4_] with *n* = 2 ^19,20^ and 3.^19^ Noteworthy, in these examples, the linkers are achiral linear molecules, with the two functional groups at the opposite sides of the chain, generating 2D layered structures. The present cations are molecular entities not yet used in any hybrid metal halide and clearly in any chiral system and resulted in a novel structural arrangement and polar interaction network. In addition, it is interesting to observe that this is the first chiral ligand where the chiral carbon does not bear an amine group but rather a hydroxyl group. The crystal structure of (*R*-AMOL)­SnI_3_ is in agreement with that of the *S* enantiomer but, as expected, showing the opposite arrangement in space (cf. Table 1). Attempts to grow single crystals of the Pb analogues were not successful. However, by looking at the powder XRD patterns of the four samples reported here and shown in Figure 3a, it can be observed that the main characteristics peaks of the (*R/S*-AMOL)­SnI_3_ are also found for the lead samples.

**Figure 3:**
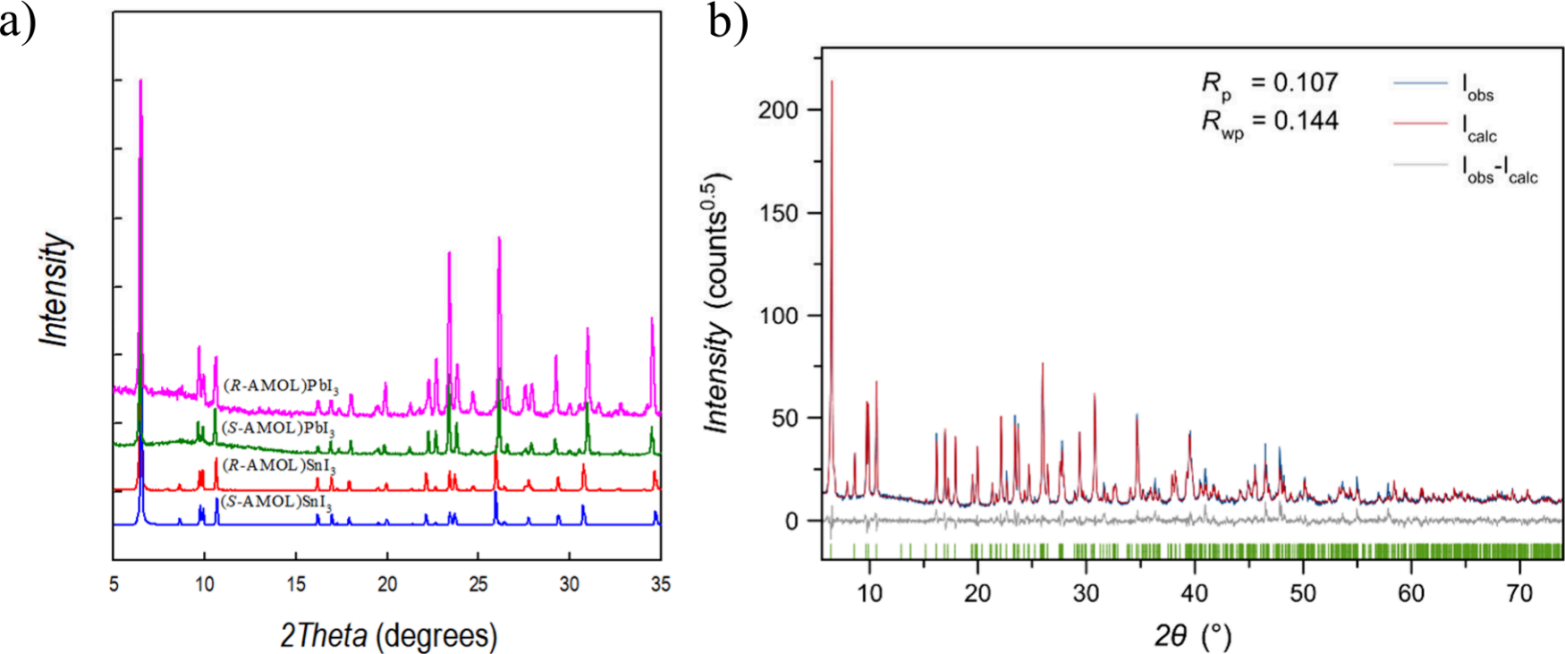
a) Powder X-ray diffraction patterns of (*R/S*-AMOL)­SnI_3_ and (*R/S*-AMOL)­PbI_3_; b) Rietveld refinement plot of the (*R*-AMOL)­PbI_3_ PXRD pattern in terms of experimental, calculated, and difference traces (blue, red, and gray, respectively). The green ticks indicate the positions of the Bragg reflections.

Accordingly, the powder patterns for (*R/S*-AMOL)­PbI_3_ were refined starting from the single-crystal data of the Sn-containing counterparts, providing a very good fit as shown, as a representative example, in Figure 3b for (*R*-AMOL)­PbI_3_. The analogous result for (*S*-AMOL)­PbI_3_ is reported in Figure S1. By looking at Table 1 it can be seen that the Pb-containing samples have a bigger lattice volume than the chiral tin iodides, with an expansion of the *a* and *c* axes and a slight contraction of the *b* axis. The general increase of the volume could be anticipated based on the ionic radii difference between Pb­(II) and Sn­(II).

The level of octahedral distortion for the 4 samples has been quantified in terms of the octahedral quadratic elongation (λ_oct_) and bond angle variance (σ^2^), as defined by Robinson et al., as well as by calculating the distortion index, *D*.^33,34^ The distortion parameter is on the order of 0.03 for all the samples, which places the present materials as intermediate distorted compounds even though such comparison is based on available data on 2D chiral perovskites, with the structure reported in this work being uncommon.^8^ Interestingly, the impact of the variation of the central metal on the distortion index is modest for these chiral 1D metal iodides, which is a different trend with respect to what has been observed in 2D chiral perovskites. As for the latter, recent works on MBA_2_PbI_4_ and MBA_2_SnI_4_ have associated the distortion increase moving from Pb to Sn to their different hard/soft behavior, recalling the Pearson’s HSAB theory.^35^ Indeed, the coordination between the hard Sn cation and the soft I anion results in weaker bonds favoring octahedral distortion and stabilizing CH−π interaction in the organic part, enhancing the overall chirality of the system. In the present compounds, however, the presence of face-sharing octahedra limits the structural degrees of freedom and could be a key factor for the similar distortion index in the two series.^23^ Moreover, the different chiral cation nature, not displaying aromatic moieties allowing for CH−π interactions, probably behaves as another factor not stabilizing a highly distorted structure. The average B–I (B = Sn and Pb) bond length increases, as expected, moving from Sn samples (∼3.20 Å) to Pb samples (∼3.27 Å). The only significant difference in the octahedral distortion parameters is related to the bond angle variance, which is more than doubled in the (*R/S*-AMOL)­PbI_3_, indicating a greater distortion in terms of bond angles in these last samples.

Optical properties of (*R/S*-AMOL)­SnI_3_ and (*R/S*-AMOL)­PbI_3_ were determined by UV–vis and CD spectroscopies. The spectra are reported in Figures 4a–d (and Figures S2, S3).

**Figure 4:**
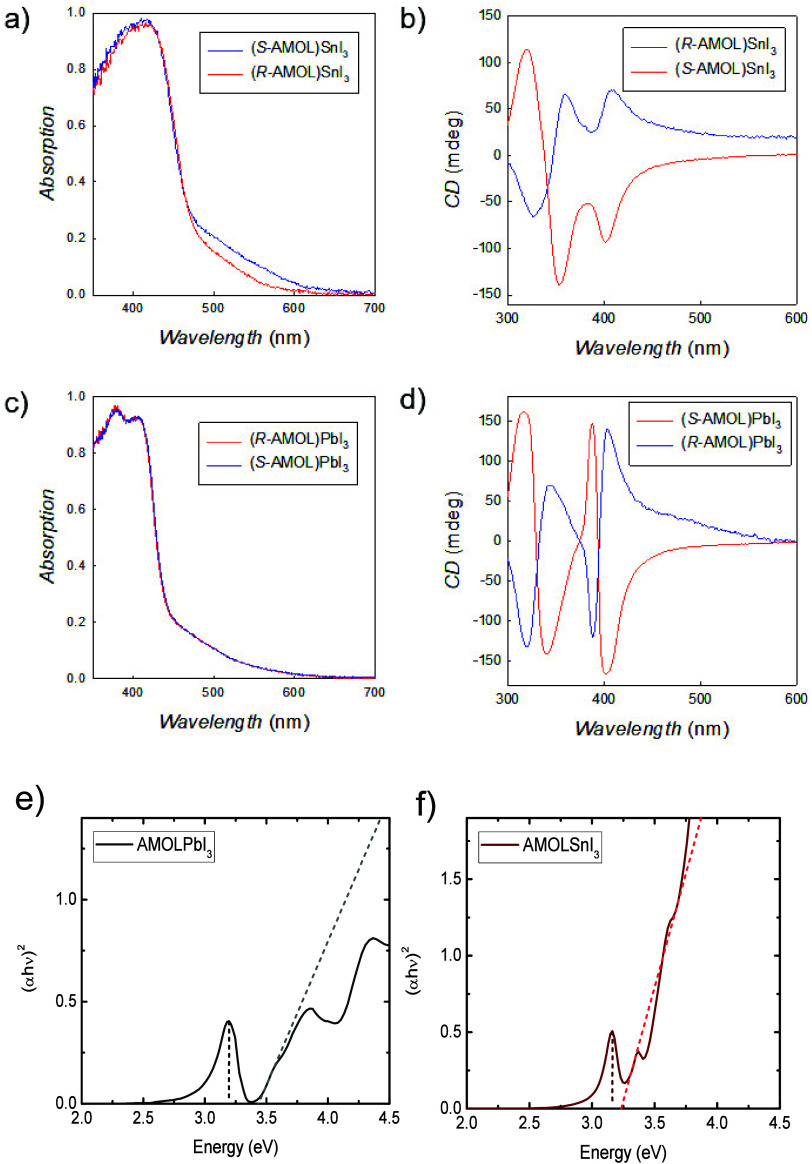
a) Absorption and b) CD spectra of (*R/S*-AMOL)­SnI_3_ and c) absorption and d) CD spectra of (*R/S*-AMOL)­PbI_3_; e, f) Tauc plot of (*R*-AMOL)­PbI_3_ and (*R*-AMOL)­SnI_3_, respectively, at 77 K. The dashed lines indicate the center of the excitonic absorption peak and the bandgap energy emerging from a linear fit of the absorption edge.

The absorption spectra for (*R/S*-AMOL)­SnI_3_, calculated from the reflectivity of powdered samples, show an absorption edge for the two chiral systems around 440 nm, while the Tauc plots are reported in Figure S2. The bandgap at room temperature was determined by the Kubelka–Munk method from diffuse reflectance spectra on drop-cast films (see Figure S3) and is estimated to be ∼2.83 eV for the direct transition. Figure 4b shows the CD spectra for the two enantiomers, which have opposite features, confirming their chirality. Three intense CD peaks with opposite sign are clearly visible in the range 300–430 nm, with this last one well corresponding to the absorption edge. For (*R/S*-AMOL)­PbI_3_ the analogous characterization provides an absorption edge around 420 nm with an extrapolated bandgap energy of 3.17 eV (see Figure S3), indicating a slight blue-shift when replacing Pb for Sn, as already observed in other metal halides even though with a smaller impact with respect to 3D and 2D metal halide perovskites.^36^

To more precisely assess the contribution of bound excitonic states on the absorption edge of these materials, we performed absorption measurements at cryogenic temperatures (77 K) on drop-cast thin films of (*R*-AMOL)­PbI_3_ and (*R*-AMOL)­SnI_3_ (Figure 4e and 4f): both materials exhibit clear excitonic absorption peaks at low temperature, respectively, at 3.2 and 3.16 eV for Pb- and Sn-containing samples. The bandgap absorption edge, retrieved by a Tauc fit of the absorption rise, is found at 3.44 and 3.23 eV, respectively. This corresponds to an estimated excitonic binding energy of 73 meV for (*R*-AMOL)­SnI_3_, consistent with no discernible excitonic absorption at room temperature, as confirmed by diffuse reflectance measurements (see Figure S3). Conversely, for (*R*-AMOL)­PbI_3_, the excitonic binding energy is 240 meV, which results in a stable excitonic population at room temperature, as corroborated by the reflectance spectrum at room temperature shown in Figure S3, where a clear excitonic peak is evident at 3.05 eV.

The CD spectra (Figure 4d) present in this case four intense and opposite peaks, with the one placed at the highest wavelength again well corresponding to the absorption edge (cf. Figure 4c). While we recognize that the mdeg value is not an absolute scale since it has a dependence on the sample amount, the present films, for both samples, of thickness around 400 nm, present one of the highest values of CD response reported to date, indicating their potential use in efficient circular polarized light photodetection. The respective chiral ligands do not show any relevant CD in the UV–vis region, as anticipated based on their molecular structure and our measurements not showing any peak. Therefore, all the chiroptically active transitions derive from a chirality transfer from the chiral bifunctional ligand to the inorganic network. It is interesting to note that an effective chirality transfer occurs also when an interaction network is established by means of −OH groups, while all the previous examples of chirality transfer to the inorganic framework were based on the hydrogen bonding through the protonated amine group. The peculiar derivative-like features of the CD signal around the band edge for all the samples suggest a lifting of the spin degeneracy within the electronic states at the edge induced by the chiral molecules defined as the Cotton effect.^37^ The chiral anisotropy factor, *g*_CD_, has been calculated from the CD measurements and resulted to be around 2 × 10^–3^ for both series of samples. Since no other chiral systems possessing the structural motif of the present samples or including a similar ligand are present in the current literature, a direct comparison of the chiroptical properties with available data is not straightforward. However, these values are higher with respect to data reported for some 2D chiral perovskites.^38^ Moreover, for 2D perovskites excitonic coupling is usually not observed in lead-based perovskites encapsulating MBA or 1-(1-naphthyl)­ethylammonium, while it appears in MBA_2_SnI_4_.^8,39^ This could be ascribed to an enhanced interaction between the organic and the inorganic moieties in the tin-based systems due to higher electronic coupling.^26^ In our 1D compounds, however, the comparable distortion and the aliphatic nature of the cation, not allowing for interactions such as the CH−π one mentioned in the literature, could induce different behavior. It can be hypothesized that the concomitant presence of two hydroxyl groups and the amino group on the cation induces strong interactions with the I atoms of the octahedra, being beneficial for the chirality transfer for both systems and resulting in their enhanced CD response displaying in all cases excitonic coupling. Overall, this complex short-range bonding pattern is capable of effectively promoting a chirality transfer to the inorganic framework as well as impacting the charge localization (see later in the text).

We also measured the photoluminescence (PL) at room temperature. Both the Pb- and Sn-based samples showed only weak PL response: the observed PL spectra (Figure S4) show a very broadband, if weak, PL signal for all samples, with an fwhm on the order of hundreds of nanometers. The PL from Pb-based samples shows two main components, centered around 500 and 700 nm. On the other hand, the Sn-based materials show a significant shift from their absorption edge, with a main peak centered around 780 nm and a broad shoulder extending to 450 nm. Such a shift in chiral Sn-based metal halides has been reported only in a previous case in the literature by our group on 2D chiral perovskites where the origin of such a phenomenon was accounted for by the presence of self-trapped excitons (STEs).^23^

To gain insight into the electronic properties of the present 1D hybrid materials, we performed DFT electronic structure analysis; see Computational Details in the SI. From the density of states (DOS) simulation, we can notice that the main contribution of the valence band (VB) is associated with the halogen and the conduction band (CB) shows a predominant contribution of the central metal (Sn or Pb), while the contribution of the organic molecules is deep in the VB and CB; see Figure 5. From a more detailed analysis of the band structure and isodensity plot of VB and CB edges, Figure 5, we can notice that the Sn-based 1D metal halides exhibit a quasi-direct bandgap, showing a band edge placed off from a high-symmetry point in the Brillouin zone: the conduction band minimum (CBM) is located at the Γ high-symmetry point, while the valence band maximum (VBM) is slightly shifted toward the X point.^40^ The band structure reveals a distinctly one-dimensional electronic character, with noticeable dispersion only along the direction of the inorganic moiety’s growth axis; see effective masses in Table S1. In the perpendicular directions, the electronic momentum is completely flat, reflecting a lack of interaction and confirming the quasi-1D nature of the material; see the band structure in Figure S7 in the SI. For the Pb-based counterpart, the bandgap is found to be direct at the Γ point.

**Figure 5:**
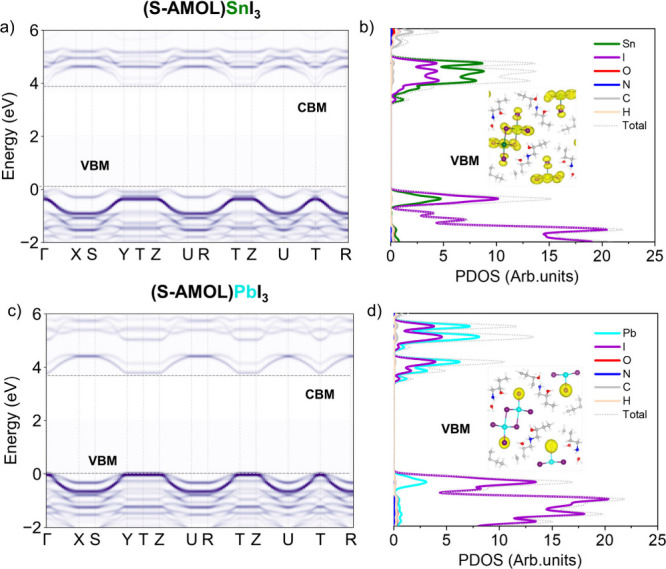
Projected band structure of the undercoordinated iodine moiety for the (a) Sn-based and (c) Pb-based chiral metal halides (*S*-enantiomer) along with the respective density of state and isodensity plots (b and d) of the VBM.

An interesting distinction from the Sn-based material is the emergence of a different character of the VBM. As we can see from the crystal structure reported in Figure 2, these systems have two different types of iodide anions: (i) the bicoordinated and (ii) the undercoordinated species. By analyzing the isodensity plots of the charge distribution associated with the VB edge state of the Sn-based material, we found a quite homogeneous contribution from all iodide atoms, while for the Pb-based species, the VB edge contribution comes from only the undercoordinated iodide; see Figure 5a and 5c.

To clearly visualize along the band structure the role of the different types of iodide, in Figure 5a and c, we illustrated the projected contributions of the undercoordinated iodine atoms to the band structure, highlighting their roles in the valence and conduction bands for the (*S*-AMOL)­SnI_3_ and (*S*-AMOL)­PbI_3_, as selected examples. In the Sn-based system (Figure 5a), both coordinated and uncoordinated iodine contribute to the valence band, emphasizing their critical role in shaping the electronic states near the Fermi level. Conversely, for the Pb-based system (Figure 5c), the valence band is primarily localized on the uncoordinated iodine, indicating a clear localization of the charge on the iodine that could in principle also lead to a shift in the electronic structure following the effect of having different metal centers. These projections provide a detailed visualization of the orbital contributions and demonstrate how iodine coordination geometry (coordinated vs uncoordinated) governs the electronic properties of these materials. Lastly, the more localized form of the charge of the valence band isodensity described in Figure 5d may be associated with the greater exciton binding energy obtained for the Pb-based system. According to Wu et al., it is well-known that the *E*_b_ rises as valence electron localization increases because of the decreased electronic screening.^41^ This distinction further underscores the differing bonding environments and electronic interactions in Sn- and Pb-based chiral low-dimensional metal halides with potential implications for their optoelectronic performance.

## Conclusions

The (2*R*,2′*R*)-1,1′-azanediylbis­(butan-2-ol) and (2*S*,2′*S*)-1,1′-azanediylbis­(butan-2-ol) bifunctional chiral ligands have been synthesized for the first time and used to prepare the 1D chiral metal halides (*R/S*-AMOL)­PbI_3_ and (*R/S*-AMOL)­SnI_3_. The presence of aminic and hydroxyl functional groups, these last on the chiral carbon, leads to the formation of an unprecedented structural arrangement in low-dimensional chiral metal halides. While this structure is found for both the Sn- and Pb-based systems, relevant differences are found in the optical response and electronic structure. While both samples show a strong CD response, centered around the absorption edge, the PL, while weak for both, is strongly red-shifted for (*R/S*-AMOL)­SnI_3_, which also displays an exciton binding energy three times lower than the Pb-counterpart. The calculation of the electronic structure of the samples shows some similarities in terms of atom contribution to the VB and CB but a significant difference arising from the nature of the iodide atoms participating in the density of states. Specifically, both bicoordinated and undercoordinated iodides contribute to the VB in the case of (*R/S*-AMOL)­SnI_3_, while for (*R/S*-AMOL)­PbI_3_ the VB is mostly localized on the axial atoms, causing a shift of the electronic structure, which can be correlated to the observed optoelectronic properties.

The present results, reporting an *ad hoc* synthesized difunctional chiral ligand leading to a novel structural family of 1D chiral metal halides, highlight the importance of extending the actual library of hybrid chiral systems in order to widen the property tuning of the chiroptical and electronic properties.

## Supplementary Material



## Notes

Materials and methods and computational details (PDF)

## References

[1] Dang Y., Liu X., Cao B., Tao X. (2021). Chiral Halide Perovskite Crystals
for Optoelectronic Applications. Matter.

[2] Dang Y., Liu X., Sun Y., Song J., Hu W., Tao X. (2020). Bulk Chiral
Halide Perovskite Single Crystals for Active Circular Dichroism and
Circularly Polarized Luminescence. J. Phys.
Chem. Lett..

[3] Long G., Sabatini R., Saidaminov M. I., Lakhwani G., Rasmita A., Liu X., Sargent E. H., Gao W. (2020). Chiral-Perovskite Optoelectronics. Nat. Rev. Mater..

[4] Coccia C., Moroni M., Malavasi L. (2023). Chiral Metal Halide
Perovskites:
Focus on Lead-Free Materials and Structure-Property Correlations. Molecules.

[5] Wei Q., Ning Z. (2021). Chiral Perovskite Spin-Optoelectronics
and Spintronics: Toward Judicious
Design and Application. ACS Materials Lett..

[6] Lu H., Wang J., Xiao C., Pan X., Chen X., Brunecky R., Berry J. J., Zhu K., Beard M. C., Vardeny Z. V. (2019). Spin-Dependent Charge Transport through
2D Chiral Hybrid
Lead-Iodide Perovskites. Sci. Adv..

[7] Yao L., Niu G., Li J., Gao L., Luo X., Xia B., Liu Y., Du P., Li D., Chen C., Zheng Y., Xiao Z., Tang J. (2020). Circularly
Polarized Luminescence
from Chiral Tetranuclear Copper­(I) Iodide Clusters. J. Phys. Chem. Lett..

[8] Lu H., Xiao C., Song R., Li T., Maughan A. E., Levin A., Brunecky R., Berry J. J., Mitzi D. B., Blum V., Beard M. C. (2020). Highly Distorted Chiral Two-Dimensional
Tin Iodide Perovskites for Spin Polarized Charge Transport. J. Am. Chem. Soc..

[9] Lin J., Chen D., Yang L., Lin T., Liu Y., Chao Y., Chou P., Chiu C. (2021). Tuning the Circular
Dichroism and Circular Polarized Luminescence Intensities of Chiral
2D Hybrid Organic–Inorganic Perovskites through Halogenation
of the Organic Ions. Angew. Chem. Int. Ed.

[10] Zhou C., Chu Y., Ma L., Zhong Y., Wang C., Liu Y., Zhang H., Wang B., Feng X., Yu X., Zhang X., Sun Y., Li X., Zhao G. (2020). Photoluminescence
Spectral Broadening, Chirality Transfer and Amplification of Chiral
Perovskite Materials (R-X- *p* -mBZA)_2_ PbBr_4_ (X = H, F, Cl, Br) Regulated by van Der Waals and Halogen
Atoms Interactions. Phys. Chem. Chem. Phys..

[11] Ma S., Jung Y.-K., Ahn J., Kyhm J., Tan J., Lee H., Jang G., Lee C. U., Walsh A., Moon J. (2022). Elucidating
the Origin of Chiroptical Activity in Chiral 2D Perovskites through
Nano-Confined Growth. Nat. Commun..

[12] Liu S., Kepenekian M., Bodnar S., Feldmann S., Heindl M. W., Fehn N., Zerhoch J., Shcherbakov A., Pöthig A., Li Y., Paetzold U. W., Kartouzian A., Sharp I. D., Katan C., Even J., Deschler F. (2023). Bright Circularly
Polarized Photoluminescence in Chiral Layered Hybrid Lead-Halide Perovskites. Sci. Adv..

[13] Yuan C., Li X., Semin S., Feng Y., Rasing T., Xu J. (2018). Chiral Lead
Halide Perovskite Nanowires for Second-Order Nonlinear Optics. Nano Lett..

[14] Trujillo-Hernández K., Rodríguez-López G., Espinosa-Roa A., González-Roque J., Gómora-Figueroa A. P., Zhang W., Halasyamani P. S., Jancik V., Gembicky M., Pirruccio G., Solis-Ibarra D. (2020). Chirality Control in White-Light
Emitting 2D Perovskites. J. Mater. Chem. C.

[15] Ren H., Wu Y., Wang C., Yan Y. (2021). 2D Perovskite Nanosheets with Intrinsic
Chirality. J. Phys. Chem. Lett..

[16] Yan L., Jana M. K., Sercel P. C., Mitzi D. B., You W. (2021). Alkyl–Aryl
Cation Mixing in Chiral 2D Perovskites. J. Am.
Chem. Soc..

[17] Moroni M., Coccia C., Malavasi L. (2024). Chiral 2D and Quasi-2D Hybrid Organic
Inorganic Perovskites: From Fundamentals to Applications. Chem. Commun..

[18] Li X., Hoffman J. M., Kanatzidis M. G. (2021). The 2D Halide Perovskite Rulebook:
How the Spacer Influences Everything from the Structure to Optoelectronic
Device Efficiency. Chem. Rev..

[19] Lemmerer A., Billing D. G. (2010). Effect of Heteroatoms
in the Inorganic–Organic
Layered Perovskite-Type Hybrids [(ZC _n_ H _2n_ NH _3_) _2_ PbI _4_ ], n = 2, 3, 4, 5, 6; Z =
OH, Br and I; and [(H _3_ NC _2_ H _4_ S _2_ C _2_ H _4_ NH _3_)­PbI _4_. CrystEngComm.

[20] Mercier N., Poiroux S., Riou A., Batail P. (2004). Unique Hydrogen Bonding
Correlating with a Reduced Band Gap and Phase Transition in the Hybrid
Perovskites (HO­(CH _2_) _2_ NH _3_) _2_ PbX _4_ (X = I, Br). Inorg.
Chem..

[21] Bouguima S., Ouahrani T., Bouheddadj A., Roux M. L., Errandonea D., Badawi M. (2021). Understanding the Optical
and Bonding Properties of
Hybrid Metal-Halide (C5H16NP) PbX4 (X = Cl, Br, I) Perovskite: A Density-Functional
Theory Study. Inorg. Chem. Commun..

[22] Zhao L., Han X., Zheng Y., Yu M.-H., Xu J. (2021). Tin-Based Chiral Perovskites
with Second-Order Nonlinear Optical Properties. Advanced Photonics Research.

[23] Coccia C., Morana M., Mahata A., Kaiser W., Moroni M., Albini B., Galinetto P., Folpini G., Milanese C., Porta A., Mosconi E., Petrozza A., De Angelis F., Malavasi L. (2024). Ligand-Induced Chirality in ClMBA _2_ SnI _4_ 2D Perovskite**. Angew. Chem. Int.
Ed.

[24] Coccia C., Moroni M., Malavasi L. (2023). Chiral Metal
Halide Perovskites:
Focus on Lead-Free Materials and Structure-Property Correlations. Molecules.

[25] Tao K., Li Q., Yan Q. (2024). 1D Tin­(II)-Based
Chiral Hybrid Perovskite Single Crystals
with Extremely Distorted Inorganic Chains for Second Harmonic Generation. Advanced Optical Materials.

[26] Fortino M., Mattoni A., Pietropaolo A. (2023). Atomistic
Modeling of Metal–Ligand
Chirality Transfer and Chiroptical Properties of Lead and Tin Hybrid
Perovskites. J. Mater. Chem. C.

[27] Coccia C., Moroni M., Treglia A., Boiocchi M., Yang Y., Milanese C., Morana M., Capsoni D., Porta A., Petrozza A., Stroppa A., Malavasi L. (2024). Unraveling the Role
of Structural Topology on Chirality Transfer and Chiroptical Properties
in Chiral Germanium Iodides. J. Am. Chem. Soc..

[28] Jiang S., Zhao P., Xing G., Kang H., Li X., Zhao T., Li B., Zhang T. (2023). Bismuth-Based Chiral
Perovskite with Different Dimensions for Second-Order Nonlinear Optical
Properties. Advanced Optical Materials.

[29] Peng H., Liu Q., Lu Y.-Z., Yang S.-J., Qi J.-C., Chen X.-G., Liao W.-Q. (2023). A Chiral
Two-Dimensional Perovskite-like Lead-Free
Bismuth­(iii) Iodide Hybrid with High Phase Transition Temperature. Chem. Commun..

[30] Rajput P. K., Poonia A. K., Mukherjee S., Sheikh T., Shrivastava M., Adarsh K. V., Nag A. (2022). Chiral Methylbenzylammonium Bismuth
Iodide with Zero-Dimensional Perovskite Derivative Structure. J. Phys. Chem. C.

[31] Wang, H. ; Li, J. ; Lu, H. ; Gull, S. ; Shao, T. ; Zhang, Y. ; He, T. ; Chen, Y. ; He, T. ; Long, G. Chiral Hybrid Germanium­(II) Halide with Strong Nonlinear Chiroptical Properties. Angew. Chem. Int. Ed 2023, 62 (41), 10.1002/anie.202309600. 37610865

[32] Eppel S., Fridman N., Frey G. (2015). Amide-Templated
Iodoplumbates: Extending
Lead-Iodide Based Hybrid Semiconductors. Cryst.
Growth Des..

[33] Robinson K., Gibbs G. V., Ribbe P. H. (1971). Quadratic Elongation: A Quantitative
Measure of Distortion in Coordination Polyhedra. Science.

[34] Baur W. H. (1974). The Geometry
of Polyhedral Distortions. Predictive Relationships for the Phosphate
Group. Acta Crystallogr. B Struct Sci..

[35] Fortino M., Mattoni A., Pietropaolo A. (2024). The Role of
Metal-Halide Bond in
the Distortions and Asymmetric Non-Covalent Interactions in Chiral
Hybrid Perovskites. J. Phys. Mater..

[36] Chiara R., Accorsi G., Listorti A., Coduri M., Coccia C., Tedesco C., Morana M., Malavasi L. (2023). Halide Alloying and
Role of Central Atom on the Structural and Optical Properties of Decylammonium
Germanium 2D Perovskites. APL Energy.

[37] Ben-Moshe A., Teitelboim A., Oron D., Markovich G. (2016). Probing the
Interaction of Quantum Dots with Chiral Capping Molecules Using Circular
Dichroism Spectroscopy. Nano Lett..

[38] Das R., Hossain M., Mahata A., Swain D., De Angelis F., Santra P. K., Sarma D. D. (2023). Unique
Chiro-Optical Properties of
the Weakly-2D (R-/S-MBA) _2_ CuBr _4_ Hybrid Material. ACS Materials Lett..

[39] Jana M. K., Song R., Liu H., Khanal D. R., Janke S. M., Zhao R., Liu C., Valy Vardeny Z., Blum V., Mitzi D. B. (2020). Organic-to-Inorganic
Structural Chirality
Transfer in a 2D Hybrid Perovskite and Impact on Rashba-Dresselhaus
Spin-Orbit Coupling. Nat. Commun..

[40] He C., Zhang C., Li J., Peng X., Meng L., Tang C., Zhong J. (2016). Direct and
Quasi-Direct Band Gap
Silicon Allotropes with Remarkable Stability. Phys. Chem. Chem. Phys..

[41] Dvorak M., Wei S.-H., Wu Z. (2013). Origin of the Variation of Exciton
Binding Energy in Semiconductors. Phys. Rev.
Lett..

