# Supplementary material for: Engineering the Electronic Structure and Optoelectronic Properties of Chiral Metal Halides through Cation Design
Source: ACS Mater Lett. 2025 Jul 18;7(8):2980–7. doi: 10.1021/acsmaterialslett.5c00666 (PMC12327265; doi:10.1021/acsmaterialslett.5c00666)
Supplement: Supplementary file 1 [file tz5c00666_si_001.pdf]

# SUPPORTING INFORMATION

# Engineering the Electronic Structure and Optoelectronic Properties of Chiral Metal Halides through Cation Design

*Clarissa Coccia,[a] Marco Moroni,[a,*] Massimo Boiocchi,[b] Marta Morana,[d] Maddalena Patrini,[e] Doretta Capsoni,[a] Alessio Porta,[a] Andera Olivati[c], Giulia Folpini,[c,d] Annamaria Petrozza,[c] Luca Gregori,[f,g] Edoardo Mosconi,[g,h] , Filippo De Angelis,[f,g,i] and Lorenzo Malavasi,[a,*]*

[a]University of Pavia, Department of Chemistry and INSTM, Via Taramelli 12, 27100, Pavia, Italy

[b]University of Pavia, Centro Grandi Strumenti, Via Bassi 21, 27100, Pavia, Italy

[c]Center for Nano Science and Technology@PoliMi, Istituto Italiano di Tecnologia 20134 Milan (Italy)

[d]Institute for Photonics and Nanotechnology, CNR – IFN, 20133 Milano

[e]University of Pavia, Department of Physics, Via Bassi 6, 27100, Pavia, Italy

[f]University of Perugia, Department of Chemistry, Biology and Biotechnology, Via Elce di Sotto 8, 06123, Perugia, Italy

[g]Computational Laboratory for Hybrid/Organic Photovoltaics (CLHYO), Istituto CNR di Scienze e Tecnologie Chimiche "Giulio Natta" (CNR-SCITEC), Via Elce di Sotto 8, 06123 Perugia, Italy

[h] Chemistry Department, College of Science, King Saud University, 11451, Riyadh, Saudi Arabia

[i]SKKU Institute of Energy Science and Technology (SIEST), Sungkyunkwan University, Suwon 440-746, South Korea

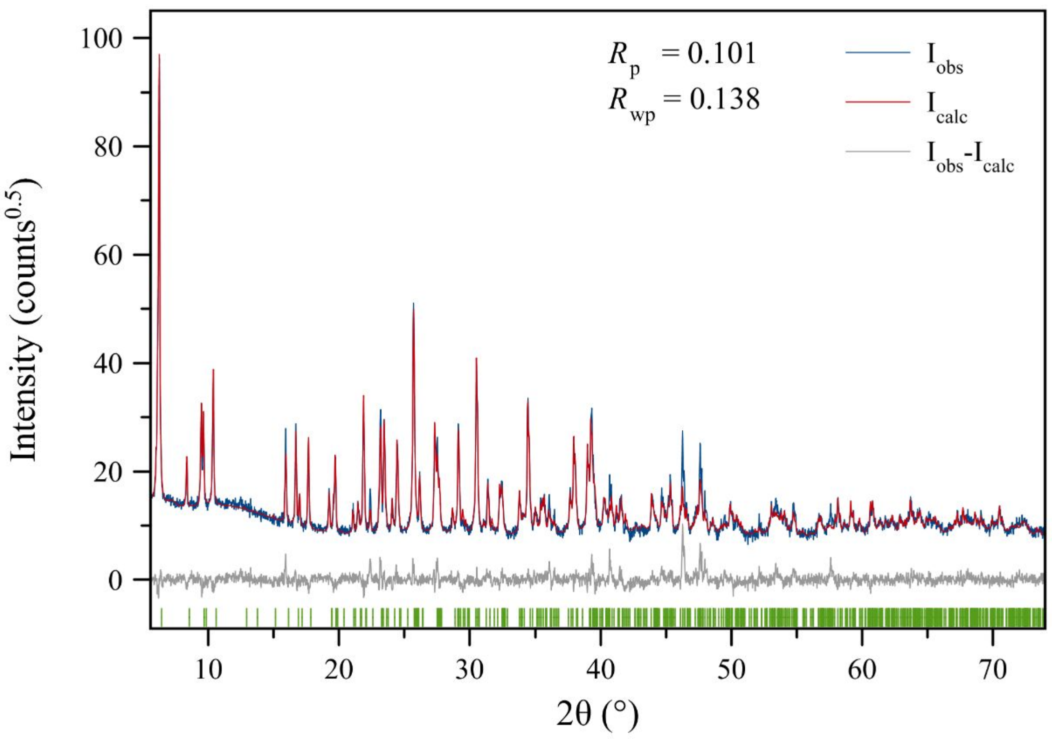


**Figure S1.** Rietveld refinement of ($S$-AMOL)$PbI_3$.

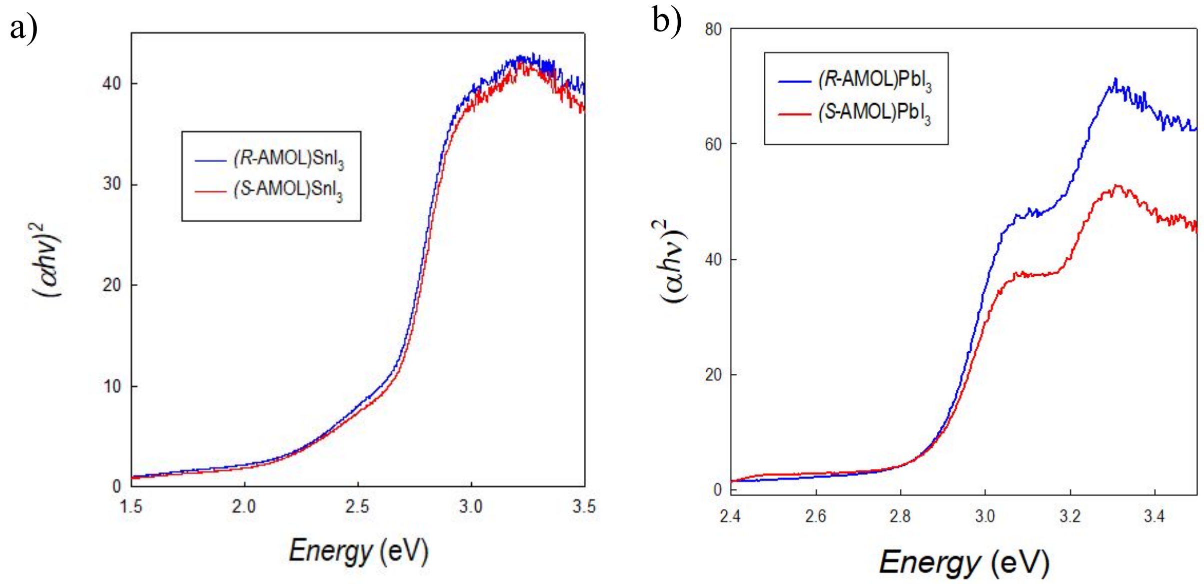


**Figure S2.** Tauc plots of a) ($R$/$S$-AMOL)$SnI_3$ and b) ($R$/$S$-AMOL)$PbI_3$.

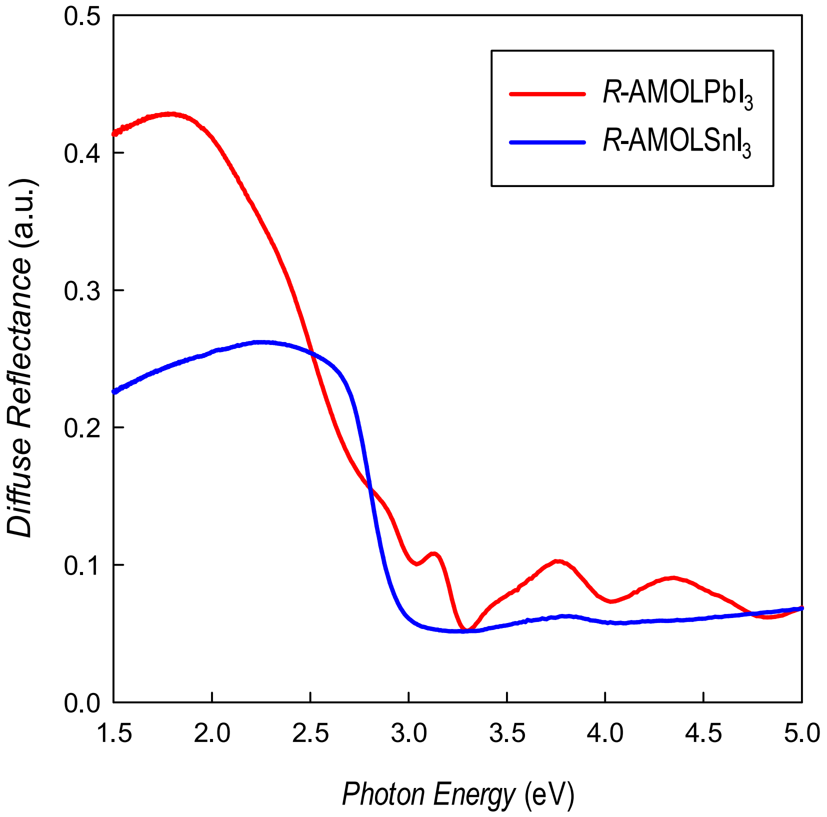


**Figure S3** Diffuse Reflectance spectra on thin films of (*R*-AMOL)$SnI_3$ and (*R*-AMOL)$PbI_3$ carried out at room temperature.

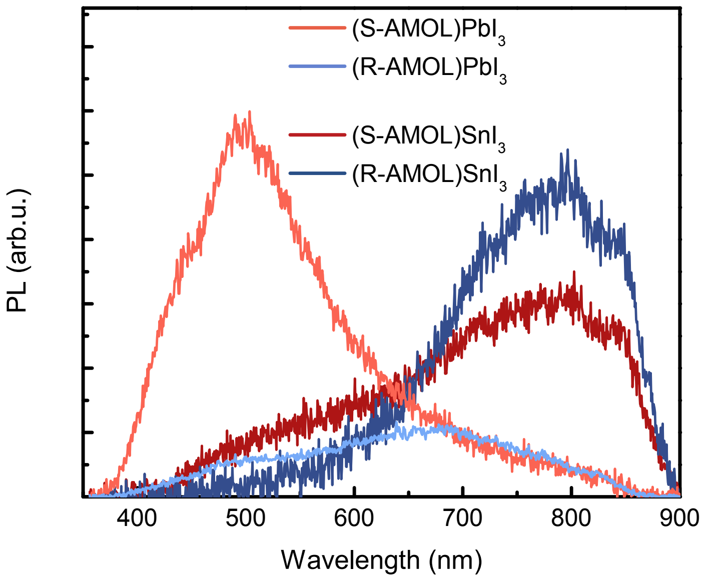


**Figure S4** PL spectra of the samples at room temperature.

# Experimental

## Synthesis of Chiral Cations

In agreement with reaction conditions, starting from (*R*) epoxide the (*R,R*) amino-diols with the same enantiomeric excess (e.e.) of starting material (> 99%) are obtained and, analogously, if the epoxide is (*S*), the (*S,S*) amino-diols will be obtained. On the other hand, the situation was more complicated if the starting material was a racemic mixture since two different diastereoisomers in racemic mixture are obtained. For this reason, we focused our attention on an enantiopure starting material for the preparation of (*R,R*) or (*S,S*) amino-diols. The representative structures of AMOL monomers and dimers are reported in Figure S5. The synthesis of dimeric and/or monomeric amino-alcohols was carried out starting from epoxides which are readily available versatile building blocks for the synthesis of many structural motifs. These compounds were easily prepared from racemic mixtures and the reactivity towards the regiospecific oxirane ring opening with soft nucleophiles is well known.[1–4] The two AMOL-monomers in enantiopure form were prepared following the literature procedures starting from the corresponding epoxide.[5] From a synthetic point of view, it was possible to prepare monomeric AMOLs and dimeric AMOLs by changing some reaction conditions. It should be emphasized that the preparation of the two enantiomers of dimeric AMOLs has been subjected of an extensive optimization work to maximize the amount of what is usually an undesired by-product. A detailed description of the synthetic procedure is reported below for the monomers and the dimers, respectively, together with spectroscopic data, including the optical rotation power. From the stereochemical point of view, the absolute configuration of starting material is the same of amino alcohol obtained following the protocols described in the literature.

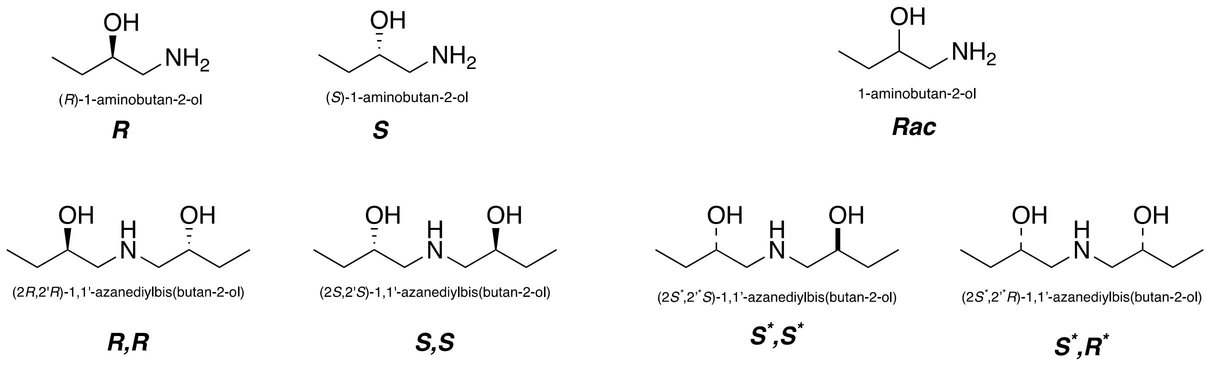


**Figure S5**. Structures of monomeric (top) and dimeric form (bottom) of amino alcohols. $S^*$,$S^*$ and $S^*$, $R^*$ are the IUPAC notation for the two pairs of diastereoisomers.

*Synthesis of (2R,2'R)-1,1'-azanediylbis(butan-2-ol) and (2S,2'S)-1,1'-azanediylbis(butan-2-ol) (AMOL-monomer)*

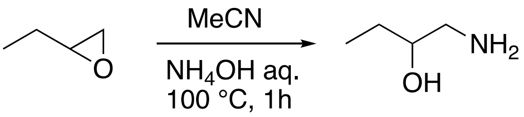


To a stirred solution of *(R/S)*-1,2-epoxybutane (1.72 mL, 20 mmol) in MeCN (4 mL) Aqueous $NH_3$ solution (10 mL, 30% w/w) was added dropwise. The resulting clear solution was heated at 100°C in a pressure tube apparatus for 1h. Volatiles were removed from the crude compound with rotary evaporator without heating the bath. The residue was then distilled bulb-to-bulb (7 mmHg, 127 °C) gave to give the two enantiomers of 1-amminobutan-2-ol as a colourless oil.

IR (liquid film): 3355 (br, NH and OH), 2931, 2861, 1579, 1076.

$^1$H NMR (400 MHz, $CDCl_3$) 3.45-3.41 (m, 1H), 2.80 (dt, $J$ =13, 4, 3 Hz, 1H, CH(*H*)N), 2.51 (dt, $J$ = 10, 5, 4 Hz, 1H, CH(H)N), 2.24 (br, 3H, $NH_2$, OH overlapped), 1.47-1.39 (m, 2H, $CH_2CH_3$), 0.94 (t, J = 7.9Hz, 3H, $CH_3$);

$^{13}$C NMR (100 MHz, $CDCl_3$) 73.4 d, 46.8 t, 27.6 t, 10.1 q.

MS-ESI: 90.09 $[M+H]^+$.

The data were the same for (*R*) and (*S*) enantiomer, ORP were reported below:

$[\alpha]^{20}_D$ = +7.6 (c 1.0, $CH_2Cl_2$) for (*R*)-1-aminobutan-2-ol.

$[\alpha]^{20}_D$ = -7.5 (c 1.1, $CH_2Cl_2$) for (*S*)-1-aminobutan-2-ol.

*Synthesis of (2R,2'R)-1,1'-azanediylbis(butan-2-ol) and (2S,2'S)-1,1'-azanediylbis(butan-2-ol) (AMOL-dimer)*

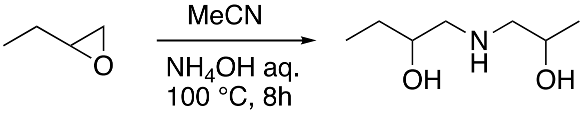


To a stirred solution of *(R/S)*-1,2-epoxybutane (1.72 mL, 20 mmol) in MeCN (4 mL) Aqueous $NH_3$ solution (1.6 Equiv., 2 mL, 30% w/w) was added dropwise. The resulting clear solution was heated at 100°C in a pressure tube apparatus for 8h. Volatiles were removed from the crude compound with rotary evaporator without heating the bath. The residue was then distilled bulb-to-bulb (7 mmHg, 127

°C) gave to remove residue of 1-amminobutan-2-ol as a colourless oil. The residue contains AMOL-dimer with more than 93% grade of purity (HESI-LC/MS).

$^{1}$H NMR (400 MHz, $CDCl_3$) δ 3.64 – 3.48 (m, 2H), 3.47 – 2.94 (m, 4H), 2.81 – 2.35 (m, 4H), 1.40 (tdd, *J* = 13.9, 10.2, 7.7 Hz, 4H), 0.91 (dd, *J* = 8.3, 6.8 Hz, 6H).

$^{13}$C NMR (101 MHz, $CDCl_3$) δ 70.9 d, 47.1 t, 28.2 t, 10.1 q.

$[\alpha]^{20}_{D}$ = +3.6 (c 1.0, $CH_2Cl_2$) for (*R*)-Dimer.
$[\alpha]^{20}_{D}$ = -3.5 (c 1.1, $CH_2Cl_2$) for (*S*)-Dimer.

*Synthesis of (R/S-AMOL)SnI$_3$ and (R/S-AMOL)PbI$_3$*

Powdered samples of (*R/S*-AMOL)$PbI_3$ and (*R/S*-AMOL)$SnI_3$ were prepared by dissolving proper amounts of $Pb(CH_3COO)_2$ or $Sn(CH_3COO)_2$ in HI at 90°C. Once the powder was dissolved in the solution, the proper amount of (*R*/*S*- AMOL) was added. After the dissolution of the latter the solution was cooled down at room temperature (25°C), favouring the precipitation of the final compounds. The synthesis of the Sn-based perovskite was carried out under an $N_2$ atmosphere, with the addition of $H_3PO_2$ as reductive reagent for the Sn(IV), while the Pb compound has been synthesised in an ambient atmosphere.

*Thin films preparation.* First, the glass substrates were washed using water, isopropanol and acetone in a sonicator for 20 min each. Then, the proper amount of perovskites were dissolved in DMF:acetone (90:10) in order to obtain a final solution 0.08 M, and the thin films were prepared on substrates by drop casting method. The films were annealed at 100°C for 10 min on a hot plate. Film thickness was measured with a mechanical profilometer (AP06 KLA Tencor).

*UV-vis-NIR spectroscopy.* Absorption and Diffuse Reflectance measurements were performed under ambient conditions by using a Varian Cary 6000i spectrophotometer equipped with a double monochromator, a 110 mm diameter integrating sphere, Si photomultiplier and InGaAs photodiode detectors. Spectral range was 190-1800 nm, in steps of 1 nm.

*UV-Vis spectroscopy.* DRS spectra were acquired in the wavelength range 300- 800 nm directly on the powders by using a Jasco V-750 spectrophotometer, equipped with an integrating sphere (Jasco ISV-922). For each spectrum, one accumulation was performed with a scan speed of 50 nm/min. The low temperature absorption measurements were acquired putting drop casted thin films samples in a nitrogen-cooled cryostat stage (Linkam) in a UV/VIS/NIR spectrophotometer Lambda 1050, Perkin Elmer.

*Circular Dichroism.* CD spectra were acquired in the wavelength range 350- 800 nm on thin films by using a Jasco J-1500 CD spectrophotometer. For each spectrum, three accumulations were performed with a scale of 200 mdeg/0.1 dOD, a bandwidth of 1 nm, and a scan speed of 50 nm/min. The $g_{CD}$ value for both samples has been calculated after normalizing the absorptions using the following formula:

$$g_{cd} = \frac{\Delta A}{A} = \left(\frac{Ellipticity}{32980}\right)/Abs$$

Where Ellipticity is expressed in millidegrees (mdeg). The effect of Linear Dichroism (LD) was eliminated by performing measurements on the thin films tilted by 90° and then rotated by 180°.

*Photoluminescence spectroscopy.* The PL spectra were acquired under excitation from the 3$^{rd}$ harmonic of a Nd:$YVO_4$ laser (Innolas Picolo, 900 ps, 1 kHz, 355 nm) keeping the samples under vacuum condition in a Linkam stage (P = 6e-3 mbar). The pump was focused on the sample with a 25 cm focal lens, to achieve a fluence of 2.2 W/cm$^2$. The PL was measured using a cooled iCCD (Andor iStar) coupled to a Shamrock monochromator, offering enhanced spectral and detection sensitivity by synchronizing the PL acquisition with the optical excitation via electronic gating (measurement window: 4 ns, integration time 10s).

*Structural determination.* Powder X-ray diffraction (PXRD) data were acquired employing a Bruker D8 Advance θ:θ diffractometer and a Si zero-background sample-holder, in the 2θ range 3.0-74.0°, with steps of 0.02° and a time *per* step of 10 s and treated taking advantage of the TOPAS-6 software. Suitable unit cell parameters were retrieved upon indexing the PXRD pattern through the Singular Value Decomposition algorithm, then performing a whole powder pattern refinement with the Le Bail method[2] (Figure S6a).[6,7] The structure was solved with the Simulated Annealing approach[3] and refined with the Rietveld method (Figure 1b of main text), describing the (*S*-/*R*-)AMOL as rigid bodies through the *z*-matrix formalism.[8,9] The background was described by means of a Chebyshev type polynomial function, and the instrumental contribution to the peak profile through the Fundamental Parameters Approach.[10] The crystallographic data are reported in table S1.

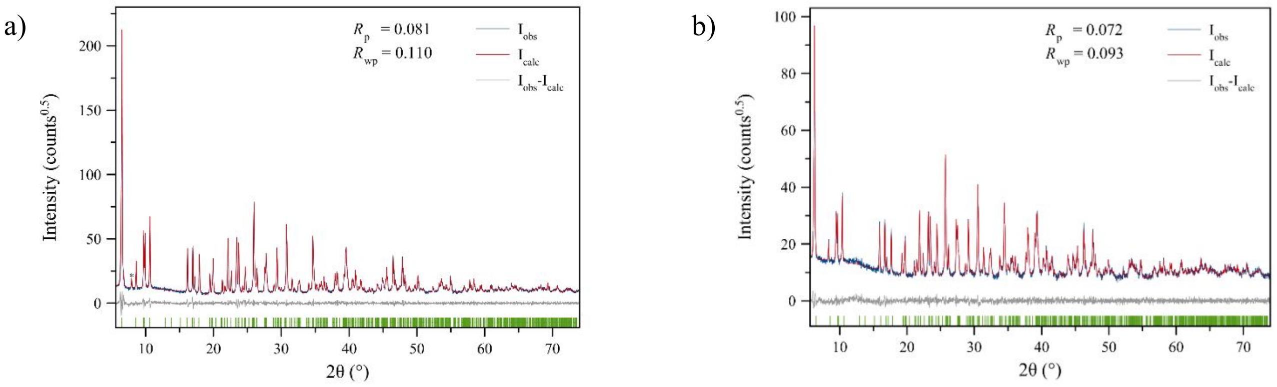


**Figure S6**. Whole powder pattern Le Bail refinement of the (*R*-AMOL)$PbI_3$ (a) and (*S*-AMOL)$PbI_3$ (b) PXRD patterns in terms of experimental, calculated and difference traces (blue, red and grey, respectively). The green ticks indicate the positions of the Bragg reflections.

## Computational Details

Density Functional Theory (DFT) calculations were performed using the Quantum ESPRESSO suite of programs and with VASP.[11–14] Initial structures for both Sn- and Pb- based perovskite models were derived from experimentally determined CIF data (CCDC Code for (*S*-AMOL)$SnI_3$: 2365047) and subsequently optimized to achieve relaxed ionic configurations. To accurately account for van der Waals interactions, we employed the PBE exchange-correlation functional augmented with DFT-D3 dispersion corrections. All the structural optimizations were carried out using QE, while VASP was used for more accurate calculations of the fine electronic properties of both materials.

Structural optimizations utilized norm-conserving pseudopotentials, explicitly including the following electronic states: I (5s, 5p), N and C (2s, 2p), H (1s), and Pb/Sn (6s, 6p, 5d).[15] A plane-wave cutoff energy of 60 Ry was applied for the wavefunctions, with a charge density cutoff of 240 Ry to ensure computational accuracy. Spin-orbit coupling (SOC) effects were incorporated post-optimization to accurately capture Rashba-type spin splitting, which is particularly significant for heavy elements such as lead. Finally, both Quantum ESPRESSO and VASP were employed for fine electronic structure calculations. Specifically, the Heyd, Scuseria, and Ernzerhof (HSE06) hybrid functional with spin-orbit coupling (SOC) addition was used, with a $6 \times 1 \times 1$ Γ-centered grid and a plane wave cut-off of 60/120 Ry for QE with 500 eV. The calculated band gaps for Sn-based perovskites were 2.64 eV using PBE, 2.31 eV with PBE-SOC, and 3.52 eV with HSE06-SOC. For Pb-based perovskites, the band gaps were slightly higher, with values of 2.85 eV (PBE), 2.16 eV

(PBE-SOC), and 3.67 eV (HSE06-SOC). These results highlight the significant impact of SOC and hybrid functionals on the electronic structure, demonstrating the need for advanced methods to accurately capture the material properties.

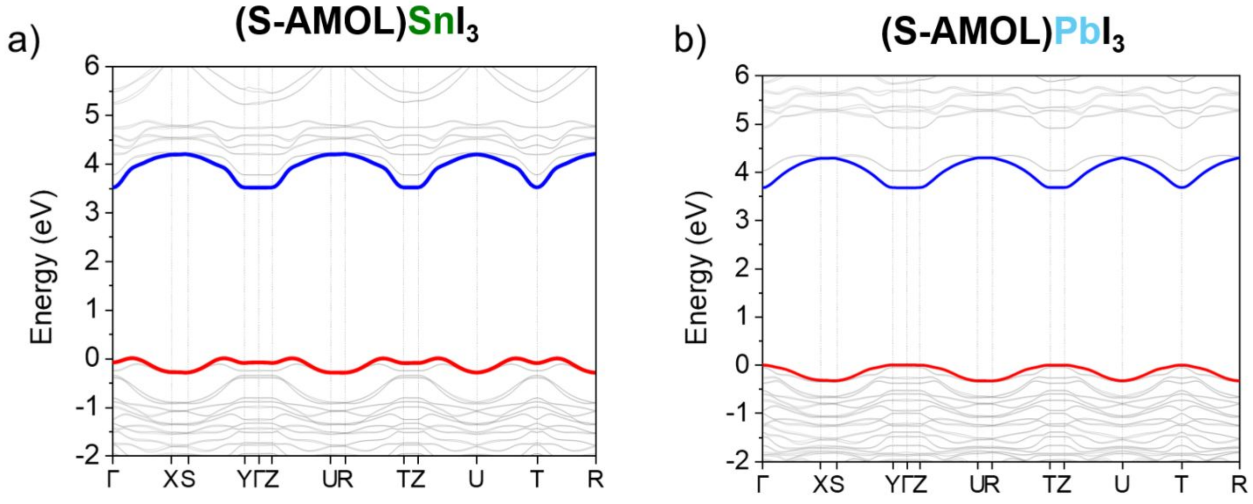


**Figure S7**: Electronic band structures of Sn-based (a) and Pb-based (b) perovskites computed using the PBE+D3 with spin-orbit coupling (SOC) and rigidly shifted to match the HSE06-SOC calculated band gap of both materials. The conduction band minimum (CBM) and valence band maximum (VBM) are highlighted in blue and red, respectively. The band structures reveal the direct band gap nature of Pb-based materials, while for Sn the valence band is found to be off the point of high symmetry leading to a quasi-indirect band gap.

**Table S1**. Hole, electron and effective masses for the investigated systems.

| | $m_h$* | $m_e$* | μ |
|---|---|---|---|
| Sn (Γ → X) | 0.59 | 0.17 | 0.13 |
| Pb (Γ → X) | 0.93 | 0.25 | 0.19 |
| Pb and Sn (Γ → Z) | ∞ | ∞ | / |

## References


(1) Tokunaga, M.; Larrow, J. F.; Kakiuchi, F.; Jacobsen, E. N. Asymmetric Catalysis with Water: Efficient Kinetic Resolution of Terminal Epoxides by Means of Catalytic Hydrolysis. *Science* **1997**, *277* (5328), 936–938. https://doi.org/10.1126/science.277.5328.936.

(2) Jacobsen, E. N. Asymmetric Catalysis of Epoxide Ring-Opening Reactions. *Acc. Chem. Res.* **2000**, *33*, 421–431. https://doi.org/10.1021/ar960061v.

(3) Saddique, F. A.; Zahoor, A. F.; Faiz, S.; Naqvi, S. A. R.; Usman, M.; Ahmad, M. Recent Trends in Ring Opening of Epoxides by Amines as Nucleophiles. *Synthetic Communications* **2016**, *46*, 831–868. https://doi.org/10.1080/00397911.2016.1170148.

(4) Shivani; Pujala, B.; Chakraborti, A. K. Zinc(II) Perchlorate Hexahydrate Catalyzed Opening of Epoxide Ring by Amines: Applications to Synthesis of ( *RS* )/( *R* )-Propranolols and ( *RS* )/( *R* )/( *S* )-Naftopidils. *J. Org. Chem.* **2007**, *72*, 3713–3722. https://doi.org/10.1021/jo062674j.

(5) Hodgson, D. M.; Humphreys, P. G.; Xu, Z.; Ward, J. G. Lithiation-Induced Migrations from Nitrogen to Carbon in Terminal Aziridines. *Angew. Chem. Int. Ed.* **2007**, *46*, 2245–2248. https://doi.org/10.1002/anie.200604920.

(6) Coelho, A. A. Indexing of Powder Diffraction Patterns by Iterative Use of Singular Value Decomposition. *J Appl Crystallogr* **2003**, *36*, 86–95. https://doi.org/10.1107/S0021889802019878.

(7) Le Bail, A.; Duroy, H.; Fourquet, J. L. Ab-Initio Structure Determination of LiSbWO6 by X-Ray Powder Diffraction. *Materials Research Bulletin* **1988**, *23*, 447–452. https://doi.org/10.1016/0025-5408(88)90019-0.

(8) Coelho, A. A. Whole-Profile Structure Solution from Powder Diffraction Data Using Simulated Annealing. *J Appl Crystallogr* **2000**, *33*, 899–908. https://doi.org/10.1107/S002188980000248X.

(9) Rietveld, H. M. A Profile Refinement Method for Nuclear and Magnetic Structures. *J Appl Crystallogr* **1969**, *2*, 65–71. https://doi.org/10.1107/S0021889869006558.

(10) Cheary, R. W.; Coelho, A. A Fundamental Parameters Approach to X-Ray Line-Profile Fitting. *J Appl Crystallogr* **1992**, *2*, 109–121. https://doi.org/10.1107/S0021889891010804.

(11) Giannozzi, P.; Baroni, S.; Bonini, N.; Calandra, M.; Car, R.; Cavazzoni, C.; Ceresoli, D.; Chiarotti, G. L.; Cococcioni, M.; Dabo, I.; Corso, A. D.; Gironcoli, S. de; Fabris, S.; Fratesi, G.; Gebauer, R.; Gerstmann, U.; Gougoussis, C.; Kokalj, A.; Lazzeri, M.; Martin-Samos, L.; Marzari, N.; Mauri, F.; Mazzarello, R.; Paolini, S.; Pasquarello, A.; Paulatto, L.; Sbraccia, C.; Scandolo, S.; Sclauzero, G.; Seitsonen, A. P.; Smogunov, A.; Umari, P.; Wentzcovitch, R. M. QUANTUM ESPRESSO: A Modular and Open-Source Software Project for Quantum Simulations of Materials. *Journal of Physics: Condensed Matter* **2009**, *21*, 395502. https://doi.org/10.1088/0953-8984/21/39/395502.

(12) Kresse, G.; Hafner, J. *Ab Initio* Molecular Dynamics for Liquid Metals. *Phys. Rev. B* **1993**, *47* (1), 558–561. https://doi.org/10.1103/PhysRevB.47.558.

(13) Kresse, G.; Furthmüller, J. Efficiency of Ab-Initio Total Energy Calculations for Metals and Semiconductors Using a Plane-Wave Basis Set. *Computational Materials Science* **1996**, *6*, 15–50. https://doi.org/10.1016/0927-0256(96)00008-0.

(14) Kresse, G.; Furthmüller, J. Efficient Iterative Schemes for *Ab Initio* Total-Energy Calculations Using a Plane-Wave Basis Set. *Phys. Rev. B* **1996**, *54*, 11169–11186. https://doi.org/10.1103/PhysRevB.54.11169.

(15) Van Setten, M. J.; Giantomassi, M.; Bousquet, E.; Verstraete, M. J.; Hamann, D. R.; Gonze, X.; Rignanese, G.-M. The PseudoDojo: Training and Grading a 85 Element Optimized Norm-Conserving Pseudopotential Table. *Computer Physics Communications* **2018**, *226*, 39–54. https://doi.org/10.1016/j.cpc.2018.01.012.
